# Peptidoglycan from Immunobiotic *Lactobacillus rhamnosus* Improves Resistance of Infant Mice to Respiratory Syncytial Viral Infection and Secondary Pneumococcal Pneumonia

**DOI:** 10.3389/fimmu.2017.00948

**Published:** 2017-08-10

**Authors:** Patricia Clua, Paulraj Kanmani, Hortensia Zelaya, Asuka Tada, A. K. M. Humayun Kober, Susana Salva, Susana Alvarez, Haruki Kitazawa, Julio Villena

**Affiliations:** ^1^Immunobiotics Research Group, Tucuman, Argentina; ^2^Laboratory of Immunobiotechnology, Reference Centre for Lactobacilli (CERELA-CONICET), Tucuman, Argentina; ^3^Food and Feed Immunology Group, Laboratory of Animal Products Chemistry, Graduate School of Agricultural Science, Tohoku University, Sendai, Japan; ^4^Livestock Immunology Unit, International Education and Research Center for Food and Agricultural Immunology (CFAI), Graduate School of Agricultural Science, Tohoku University, Sendai, Japan; ^5^Institute of Applied Biochemistry, National University of Tucumán, Tucuman, Argentina

**Keywords:** immunobiotics, peptidoglycan, toll-like receptor 3, viral immunity, *Streptococcus pneumoniae*, respiratory syncytial virus

## Abstract

Several research works have demonstrated that beneficial microbes with the capacity to modulate the mucosal immune system (immunobiotics) are an interesting alternative to improve the outcome of bacterial and viral respiratory infections. Among the immunobiotic strains with the capacity to beneficially modulate respiratory immunity, *Lactobacillus rhamnosus* CRL1505 has outstanding properties. Although we have significantly advanced in demonstrating the capacity of *L. rhamnosus* CRL1505 to improve resistance against respiratory infections as well as in the cellular and molecular mechanisms involved in its beneficial activities, the potential protective ability of this strain or its immunomodulatory cellular fractions in the context of a secondary bacterial pneumonia has not been addressed before. In this work, we demonstrated that the nasal priming with non-viable *L. rhamnosus* CRL1505 or its purified peptidoglycan differentially modulated the respiratory innate antiviral immune response triggered by toll-like receptor 3 activation in infant mice, improving the resistance to primary respiratory syncytial virus (RSV) infection, and secondary pneumococcal pneumonia. In association with the protection against RSV-pneumococcal superinfection, we found that peptidoglycan from *L. rhamnosus* CRL1505 significantly improved lung CD3^+^CD4^+^IFN-γ^+^, and CD3^+^CD4^+^IL-10^+^ T cells as well as CD11c^+^SiglecF^+^IFN-β^+^ alveolar macrophages with the consequent increases of IFN-γ, IL-10, and IFN-β in the respiratory tract. Our results also showed that the increase of these three cytokines is necessary to achieve protection against respiratory superinfection since each of them are involved in different aspect of the secondary pneumococcal pneumonia that have to be controlled in order to reduce the severity of the infectious disease: lung pneumococcal colonization, bacteremia, and inflammatory-mediated lung tissue injury.

## Introduction

Respiratory viral attack often result in mild to moderate infection; however, life-threatening disease can occur in high-risk populations such as infants, elderly, and immunocompromised hosts. Moreover, secondary bacterial pneumonia is an important complication responsible for high morbidity and mortality during epidemic and pandemic viral respiratory infections in infants and children (1–3). It was demonstrated that secondary bacterial respiratory infections are caused primarily by *Streptococcus pneumoniae*. The majority of the clinical observations and experiments in animal models have focused in post-influenza pneumococcal pneumonia (4). In fact, numerous studies have investigated how primary influenza virus (IFV) infection enhances the susceptibility to secondary pneumococcal disease, by increasing bacterial attachment and colonization, disrupting epithelial barriers, and altering the innate immune response in the respiratory tract (3). Although IFV and *S. pneumoniae* interaction has been extensively studied because of its great impact in the severity of respiratory infections, other viruses like the Respiratory Syncytial Virus (RSV) have been associated to an increased susceptibility to secondary pneumococcal pneumonia.

Clinical and epidemiologic data suggest that RSV is linked to increases in the frequency (5) and severity (6) of pneumococcal disease. It was also demonstrated that mice infected with RSV before pneumococcal challenge as well as mice infected with both respiratory pathogens simultaneously showed enhanced lung alterations and elevated levels of bacteremia (7, 8). Mechanisms underlying pneumococcal superinfection include RSV-induced local destruction of the epithelium and respiratory ciliary dyskinesia that impairs mucociliary clearance in the airways (8). Elevated pneumococcal adherence to the respiratory tract epithelium is also considered one of the mechanisms facilitating *S. pneumoniae* infection. It was reported that intercellular adhesion molecule 1 (ICAM-1), carcinoembryonic adhesion molecule 1 (CEACAM1), and platelet activating factor receptor (PAF) are upregulated by RSV infection in respiratory epithelial cells, which are molecules used by pneumococci for colonization (9). Moreover, *in vitro* experiments with HEp-2 cells (human nasopharyngeal), A549 cells (pneumocyte type II), or human airway epithelial cell primary cultures showed that RSV virions enhance pneumococcal adherence through the expression of the viral G protein in epithelial surfaces that serve as an adhesion molecule for pneumococci (7, 8, 10). Surprisingly, transcriptomic analysis performed by Smith et al. (8) showed that the direct interaction between RSV and *S. pneumoniae* alters bacterial gene expression. The work demonstrated that the pneumococcal penicillin-binding protein 1a binds RSV G protein and that this interaction alters *S. pneumoniae* transcriptome increasing the expression of the virulence factors pneumolysin and neuraminidase A/B. These results indicate that complex interactions exist between RSV, *S. pneumoniae*, and host, which must be fully characterized in order to reduce the severity and mortality of respiratory superinfections caused by these pathogens.

During the last years, several research works have demonstrated that beneficial microbes with the capacity to modulate the mucosal immune system (immunobiotics) are a potential alternative to improve the outcome of bacterial (11) and viral (12) respiratory infections. Among the immunobiotic strains with the capacity of beneficially modulate respiratory immunity, our research group has demonstrated that *Lactobacillus rhamnosus* CRL1505 has outstanding properties. Nasal priming with *L. rhamnosus* CRL1505 is able to significantly increase the resistance against the respiratory pathogens *S. pneumoniae*, IFV, or RSV (13–15). We have also reported that immunobiotic *L. rhamnosus* CRL1505 is able to differentially regulate the levels and kinetics of respiratory inflammatory cells and cytokines in mice after activation of Toll-like receptor 3 (TLR3) by the nasal administration of poly(I:C), or after the challenge with RSV or IFV (13, 14). This beneficial regulation of virus-triggered inflammatory response in the respiratory tract by the CRL1505 strain correlated with a significant reduction in lung damage and improved survival of infected mice (13, 14). Of interest, we have demonstrated that viability of the immunobiotic strain is not necessary to achieve the protective effect. In fact, protection against RSV or *S. pneumoniae* infections can be improved by nasal administration of non-viable *L. rhamnosus* CRL1505 (13) or its peptidoglycan (15), respectively. Although we have significantly advanced in demonstrating the capacity of *L. rhamnosus* CRL1505 to improve resistance against respiratory infections as well as in the cellular and molecular mechanisms involved in its beneficial activities (12), the potential protective ability of this strain or its immunomodulatory cellular fractions in the context of a secondary bacterial pneumonia has not been addressed before.

We hypothesized that the effect of immunobiotics or their immunomodulatory cellular fractions in the respiratory antiviral innate immune response could beneficially influence the resistance to secondary bacterial infections. Therefore, in the present study, we investigated how the exposure of infant mice to the nasal priming with non-viable *L. rhamnosus* CRL1505 or its peptidoglycan influences the respiratory innate immune response triggered by TLR3 activation, the susceptibility to primary RSV infection, and the resistance to secondary pneumococcal pneumonia. We demonstrated that peptidoglycan from immunobiotic *L. rhamnosus* CRL1505 improves respiratory antiviral innate immune response, reduces bacterial transmigration across the lung, and limits pulmonary inflammatory damage caused by *S. pneumoniae* after the challenge with poly(I:C) or the infection with RSV.

## Materials and Methods

### Microorganisms and Peptidoglycan

*Lactobacillus rhamnosus* CRL1505 was obtained from the CERELA culture collection (Chacabuco 145, San Miguel de Tucumán, Argentina). The culture was kept freeze-dried. For experiments the culture was rehydrated using a medium containing 15 g of peptone, 10 g tryptone, and 5 g of meat extract in 1 l of distilled water, pH 7. Then, lactobacilli were cultured for 12 h at 37°C (final log phase) in Man–Rogosa–Sharpe broth (MRS, Oxoid). The bacteria were harvested by centrifugation at 3,000 × *g* for 10 min, washed three times with sterile 0.01 mol/l phosphate buffer saline (PBS, pH 7.2), and resuspended in sterile PBS. Non-viable *L. rhamnosus* CRL1505 (HK1505) was obtained as described previously (13). Bacteria were killed by tyndallization in a water bath at 80°C for 30 min, and the lack of bacterial growth was confirmed using MRS agar plates. Peptidoglycan from *L. rhamnosus* CRL1505 (PG1505) was obtained as described previously (15). Briefly, the bacterium was grown in MRS broth for 18 h at 37°C, washed three times with sterile PBS, and lyophilized. Lactobacilli were resuspended in sterile water (0.1 g/ml) and lysed by sonication in an Ultrasonic Homogenizer (Cole Parmer) with cycles of 2.5 min and amplitude of 70%. The cell wall obtained was delipidated by successive refluxing with methanol, methanol–chloroform (1:1), and chloroform. The delipidated preparation was resuspended in Tris–HCl buffer 50 µM (pH 7.5) and treated with bovine pancreatic DNAse I (Sigma) (50 µg/ml) and ribonuclease A (Sigma) (100 µg/ml) at 37°C, 4 h. Finally, cell wall was treated with 50% hydrogen chloride at 4°C for 20 h. The PG1505 obtained was washed with sterile water, adjusted to pH 7.2, and lyophilized until use.

### Animals and Feeding Procedures

Infant (3-week-old) BALB/c mice were obtained from the closed colony kept at CERELA (San Miguel de Tucumán, Argentina). They were housed in plastic cages at room temperature. Mice were housed individually during the experiments and the assays for each parameter studied were performed in 5–6 mice per group for each time point. HK1505 was nasally administered to infant mice for 2 consecutive days at a dose of 10^8^ cells/mouse/day in 50 µl of PBS (13). PG1505 was nasally administered to infant mice for 2 consecutive days at a dose of 8 µg/ml, in 50 µl of PBS (15). Anesthesia was not necessary for HK1505 or PG1505 administration since mice did not show any sing of discomfort. The treated groups and the untreated control group were fed a conventional balanced diet *ad libitum*. This study was carried out in strict accordance with the recommendations in the Guide for the Care and Use of Laboratory Animals of the Guidelines for Animal Experimentation of CERELA and all efforts were made to minimize suffering.

### Intranasal Administration of Poly(I:C)

**Figure 1:**
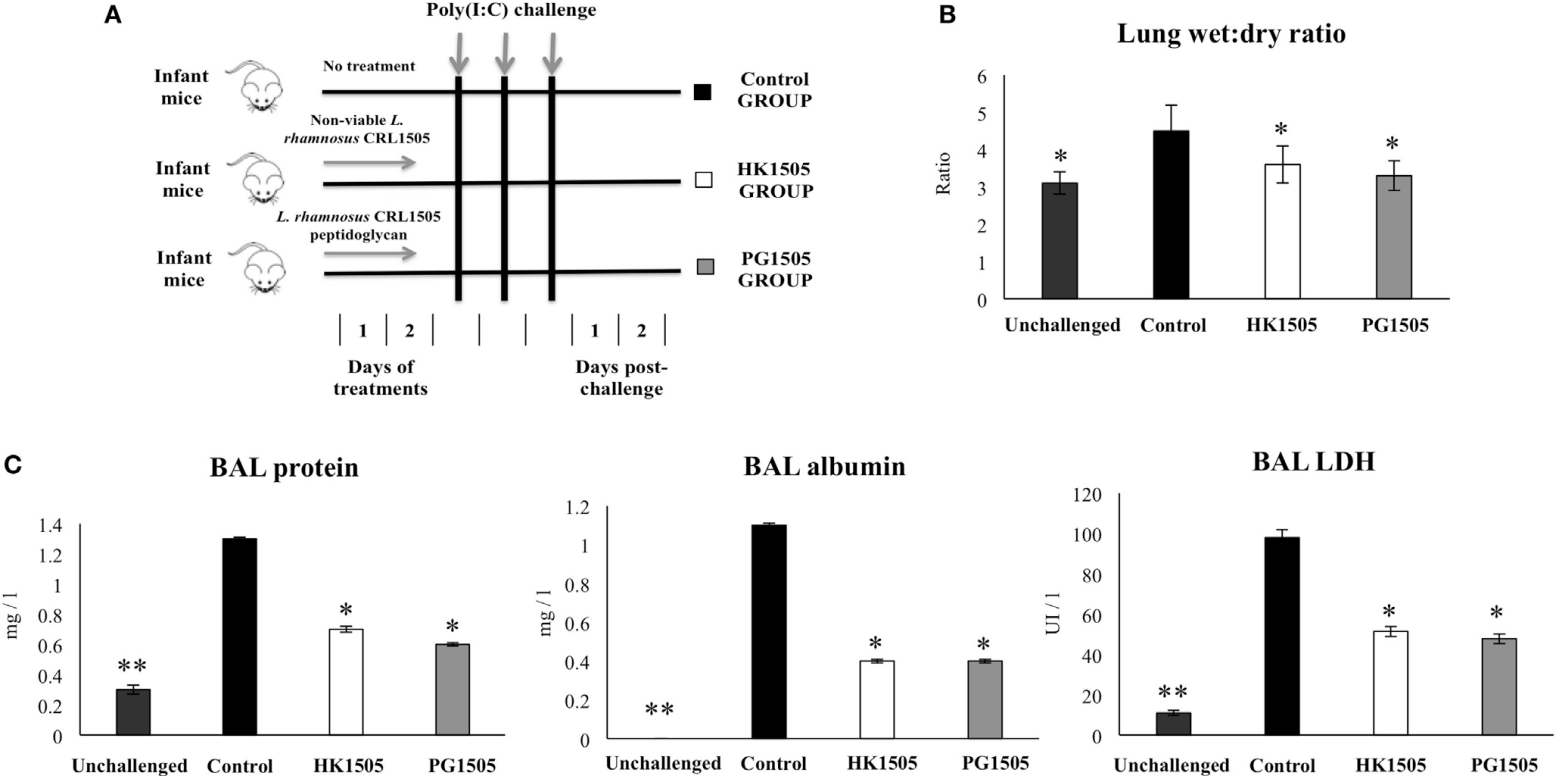
Effect of non-viable *Lactobacillus rhamnosus* CRL1505 (HK1505) and its peptidoglycan (PG1505) on lung tissue damage induced by the nasal administration of the viral pathogen-associated molecular pattern poly(I:C). Infant mice were nasally primed with HK1505 or PG1505 during two consecutive days and then challenged with three once-daily doses of poly(I:C) **(A)**. Non-treated infant mice challenged with poly(I:C) were used as controls. 2 days after the last poly(I:C) administration lung wet:dry weight ratio **(B)**, lactate dehydrogenase (LDH) activity and, albumin and protein concentrations in bronchoalveolar lavages (BAL) **(C)** were determined. The results represent data from three independent experiments. Significant differences between treated and control groups, *P < 0.05, **P < 0.05.

Administration of the viral pathogen molecular pattern poly(I:C) was performed on day 3, after the 2-day treatments with HK1505 or PG1505 as shown in Figure 1A. Mice were lightly anesthetized and 100 µl of PBS, containing 250 µg poly(I:C) (equivalent to 10 mg/kg body weight), was administered dropwise, *via* the nares (13, 16, 17). Control animals received 100 µl of PBS. Mice received three doses of poly(I:C) or PBS with 24 h rest period between each administration.

### RSV Infection

Human RSV strain A2 was grown in Vero cells as described previously (13, 16). Briefly, Vero cells were infected with RSV at a multiplicity of infection of 1 in 5 ml of Dulbecco’s modified Eagle’s medium (DMEM). Cells were infected for 3 h at 37°C, 5% CO_2_. After infection, 7 ml of DMEM with 10% fetal bovine serum (Sigma, Tokyo, Japan), 0.1% penicillin–streptomycin (Pen/Strep) (Sigma, Tokyo, Japan), and 0.001% ciprofloxacin (Bayer) was added to the flask, and cells were incubated until extensive syncytium formation was detected. Then, Vero cells were scraped and sonicated three times, 5 s per time, at 25 W on ice. Cell debris was removed by centrifugation at 700 g for 10 min at 4°C. Virus supernatant was sucrose density gradient purified and stored in 30% sucrose at −80°C. For *in vivo* infection, mice were lightly anesthetized with isoflurane and intranasally challenged with 3.1 × 10^6^ PFU of RSV (13, 16). Viral challenge was performed on day 3, after the 2-day treatments with HK1505 or PG1505. Lung RSV titers and tissue damage were evaluated during 5 days after viral infection.

For the evaluation of viral infection, the RSV immunoplaque assay was performed as described previously (13, 16). In brief, lung tissue was removed from infant mice and stored in 30% sucrose for plaque assay. Lungs were homogenized using a pellet pestle and centrifuged at 2,600 × *g* for 10 min at 4°C to clarify supernatant. Serial dilutions of lung tissue-clarified supernatants were added into fresh Vero cells monolayers, and incubated at 37°C, 5% CO_2_ for 3 h. All samples were run in triplicate. After incubation and removal of supernatant, 1 ml of fresh DMEM medium containing 10% FBS, 0.1% Pen/Strep, and 0.001% ciprofloxacin was added to monolayers. When extensive syncytia developed, monolayers were fixed with 1 ml of ice-cold acetone:methanol (60:40). Then, well were treated with primary RSV anti-F (clones 131-2A; Chemicon) and anti-G (Mouse monoclonal [8C5 (9B6)] to RSV glycoprotein, Abcam) antibodies for 2 h, followed by secondary horseradish peroxidase anti-mouse immunoglobulin antibody (Anti-mouse IgG, HRP-linked Antibody #7076, Cell signaling Technology) for 1 h. Plates washed twice with PBS containing 0.5% Tween 20 (Sigma) after each antibody incubation step. Individual plaques were developed using a DAB substrate kit (ab64238, Abcam) following manufacture’s specifications. Results were expressed as log10 PFU/g of lung.

### *S. pneumoniae* Secondary Infection

*Streptococcus pneumoniae* serotype 6B (ANLIS, Argentina) was obtained from the respiratory tract of a patient from the Children’s Hospital, Tucuman, Argentina. Pneumococci were grown on blood agar for 18 h. Colonies were suspended in Todd Hewitt broth (Oxoid), incubated overnight at 37°C, harvested, and washed with sterile PBS. Cell density was adjusted to 4 × 10^7^ CFU/ml.

Challenge with pneumococci was performed 1, 3, and 5 days after the last administration of poly(I:C) (data not shown), and a higher susceptibility to bacterial infection was found when the challenge was performed 5 days after TLR3 activation. In addition, several published articles performed bacterial infection 5–7 days after respiratory viral challenge considering that viral load and cytokine environment are different in the earlier and later stages of viral infection (4, 18). Then, challenge with pneumococci was also performed 5 days after the infection with RSV. HK1505- and PG1505-treated as well as control infant mice were challenged intranasally with the pathogen by dripping 25 µl of inoculums containing 10^3^, 10^4^, 10^6^, or 10^7^ CFU (log phase) in PBS into each nostril.

Treated and control mice were sacrificed 2 days after *S. pneumoniae* infection. Lungs were excised, weighed, and homogenized in sterile peptone water. Homogenates were diluted appropriately, plated in duplicate on blood agar and incubated for 18 h at 37°C. *S. pneumoniae* was identified by standard techniques and the results were expressed as log of CFU/g of lung. Bacteremia was monitored by blood samples obtained by cardiac puncture which were plated on blood agar. Results were reported as negative or positive hemocultures.

### Blocking Experiments

In order to evaluate the role of IFN-β, IFN-γ, and IL-10 in the immunoprotective effect of HK1505 and PG1505, anti-IFN-β, anti-IFN-γ, and anti-IL-10 receptor (IL-10R) blocking antibodies were used (16). Different groups of mice were nasally primed with HK1505 or PG1505 for 2 days and then challenged with poly(I:C) for 3 days as described above. On days 2 and 4 after poly(I:C) challenge, mice were nasally treated with 50 μg of purified IFN-β (LEAF™ Purified anti-mouse IFN-β antibody, #519202 BioLegend), purified anti-IFN-γ (LEAF™ Purified anti-mouse IFN-γ antibody, #505706 BioLegend), or anti-IL-10R (LEAF™ Purified anti-mouse IL-10R antibody, #112708, BioLegend, Tokyo, Japan) antibodies or 250 μg isotype control antibodies (LEAF™ Purified Rat IgG1, Isotype Ctrl, BioLegend). Twelve hours later mice were challenged with *S. pneumoniae* (Figure S1 in Supplementary Material). The efficiency of blocking antibodies was determined by evaluating serum and respiratory concentration of IFN-β, IFN-γ, or IL-10 12 h after the last administration.

### Lung Injury Parameters

Bronchoalveolar lavages (BAL) samples were obtained as described previously (13, 16). Briefly, the trachea was exposed and intubated with a catheter, and two sequential lavages were performed in each mouse by injecting sterile PBS. The recovered fluid was centrifuged for 10 min at 900 × *g*; and frozen at −70°C for subsequent analyses.

Protein and albumin content, a measure to quantitate increased permeability of the bronchoalveolar–capillarity barrier, and lactate dehydrogenase (LDH) activity, an indicator of general cytotoxicity, were determined in the acellular BAL fluid. Protein content was measured by the bicinchoninic (BCA) protein assay (Pierce Biotechnology Inc., Rockford, IL, USA). Albumin content was determined colorimetrically based on albumin binding to bromcresol green using an albumin diagnostic kit (Wiener Lab, Buenos Aires, Argentina). LDH activity, expressed as units per liter of BAL fluid, was determined by measuring the formation of the reduced form of nicotinamide adenine dinucleotide using the Wiener reagents and procedures (Wiener Lab). Lung wet:dry weight ratio was measured as previously described (13, 17). Wet:dry weight ratio was calculated as an index of intrapulmonary fluid accumulation, without correction for blood content.

Histopathological examination was also performed in order to further evaluate tissue damage. Lungs were aseptically removed, fixed in 4% formalin, and embedded in histowax (Leica Microsystems). Histopathological assessment was performed on 5-µm tissue sections stained with hematoxylin–eosin. At least four tissue sections from various areas of the lung of each mouse in all experimental groups were examined.

### Cytokine Concentrations in Serum and BAL

Tumor necrosis factor (TNF)-α (Mouse TNF-alpha Quantikine enzyme-linked immunosorbent assay (ELISA) Kit, sensitivity: 7.2 pg/ml), interferon (IFN)-α (Mouse IFN-alpha ELISA Kit, sensitivity: 12.5 pg/ml), IFN-β (Mouse IFN-beta ELISA Kit, sensitivity: 15.5 pg/ml), IFN-γ (Mouse IFN-gamma Quantikine ELISA Kit, sensitivity: 2 pg/ml), interleukin (IL)-6 (Mouse IL-6 Quantikine ELISA Kit, sensitivity: 1.8 pg/ml), IL-8 (Mouse IL-8 Quantikine ELISA Kit, sensitivity: 2 pg/ml), IL-10 (Mouse IL-10 Quantikine ELISA Kit, sensitivity: 5.2 pg/ml), and monocyte chemoattractant protein (MCP)-1 (Mouse/Rat CCL2/JE/MCP-1 Quantikine ELISA Kit, sensitivity: 2 pg/ml) concentrations in serum and BAL were measured with commercially available ELISA technique kits following the manufacturer’s recommendations (R&D Systems, MN, USA).

### Lung Cells Preparation and Flow Cytometry Studies

Single lung cells from mice were prepared as previously described (13, 17). Lungs were removed, finely minced, and incubated for 90 min with 300 U of collagenase (Yakult Honsha Co., Tokyo, Japan) in 15 ml of RPMI 1640 medium (Sigma, Tokyo, Japan). To dissociate the tissue into single cells, collagenase-treated minced lungs were gently tapped into a plastic dish. After removal of debris, erythrocytes were depleted by hypotonic lysis. The cells were washed with RPMI medium supplemented with 100 U/ml of penicillin and 100 mg/ml of streptomycin and then resuspended in a medium supplemented with 10% heat-inactivated fetal calf serum. Cells were counted using Trypan Blue exclusion and then resuspended at an appropriate concentration of 5 × 10^6^ cells/ml.

Lung cell suspensions were pre-incubated with anti-mouse CD32/CD16 monoclonal antibody (Fc block) for 15 min at 4°C. Cells were incubated in the antibody mixes for 30 min at 4°C and washed with FACS buffer. Then, cells were stained with fluorochrome-conjugated antibodies against CD3, CD4, CD8, CD11c, CD11b, CD103, MHC-II, IFN-γ, IL-10, sialic acid-binding immunoglobulin-like lectin F (SiglecF) (BD Bioscience), IFN-β, and CD45 (eBioscience). Cells were then acquired on a BD FACSCalibur™ flow cytometer (BD Biosciences) and data were analyzed with FlowJo software (TreeStar). The total number of cells in each population was determined by multiplying the percentages of subsets within a series of marker negative or positive gates by the total cell number determined for each tissue (13, 17).

### Statistical Analysis

Experiments were performed in triplicate and results were expressed as mean ± SD. After verification of the normal distribution of data, 2-way ANOVA was used. Tukey’s test (for pairwise comparisons of the means) was used to test for differences between the groups. Differences were considered significant at *P* < 0.05.

## Results

### Nasally Administered Peptidoglycan from *L. rhamnosus* CRL1505 Reduces Poly(I:C)-Induced Lung Injuries

We have previously demonstrated that nasal administration of non-viable *L. rhamnosus* CRL1505 (HK1505) reduced lung injuries triggered by TLR3 activation (13). Here, we aimed to evaluate the effect of the nasal priming with the peptidoglycan from *L. rhamnosus* CRL1505 (PG1505) on the immune response triggered by the viral pathogen-associated molecular pattern poly(I:C) and compare it with the effect induced by HK1505. For this purpose, infant mice were treated with PG1505 and then challenged with poly(I:C) as shown in the experimental protocol of Figure 1A. HK1505 treatment was used as a positive control. Lung injury was studied on day 2 post-challenge as we described previously (13, 17). An altered lung wet:dry weight ratio (Figure 1B), as well as increased levels of LDH activity and protein and albumin concentrations (Figure 1C) were found in BAL samples of poly(I:C)-treated infant mice, indicating local cellular damage and impairment of the alveolar-capillary barrier. Nasally administered HK1505 or PG1505 did not induce significant changes in the BAL biochemical parameters evaluated before poly(I:C) administration (data not shown). Both, HK1505 or PG1505 significantly diminished wet:dry weight ratio and the three biochemical parameters evaluated in BAL after poly(I:C) challenge (Figure 1). HK1505 and PG1505 were equally effective to reduce the lung alterations induced by TLR3 activation.

### Nasally Administered Peptidoglycan from *L. rhamnosus* CRL1505 Beneficially Modulates Immune Response Triggered by Poly(I:C) Challenge

**Figure 2:**
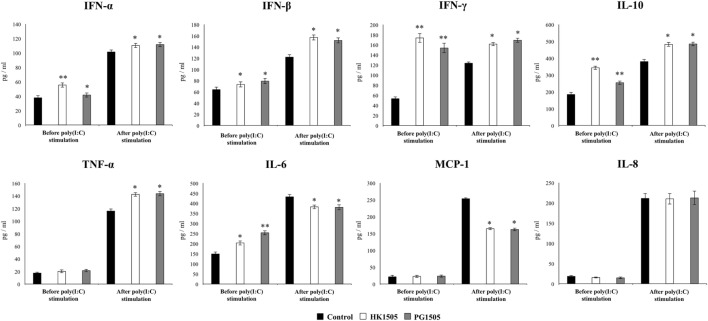
Effect of non-viable *Lactobacillus rhamnosus* CRL1505 (HK1505) and its peptidoglycan (PG1505) on respiratory cytokines before and after the nasal administration of the viral pathogen-associated molecular pattern poly(I:C). Infant mice were nasally primed with HK1505 or PG1505 during two consecutive days and then challenged with three once-daily doses of poly(I:C). Non-treated infant mice challenged with poly(I:C) were used as controls. Before and 2 days after poly(I:C) administration, the levels of tumor necrosis factor (TNF)-α, interferon (IFN)-α, IFN-β, IFN-γ, interleukin (IL)-6, IL-8, IL-10, and monocyte chemoattractant protein-1 (MCP-1) were determined in bronchoalveolar lavages. The results represent data from three independent experiments. Significant differences between treated and control groups, *P < 0.05.

**Figure 3:**
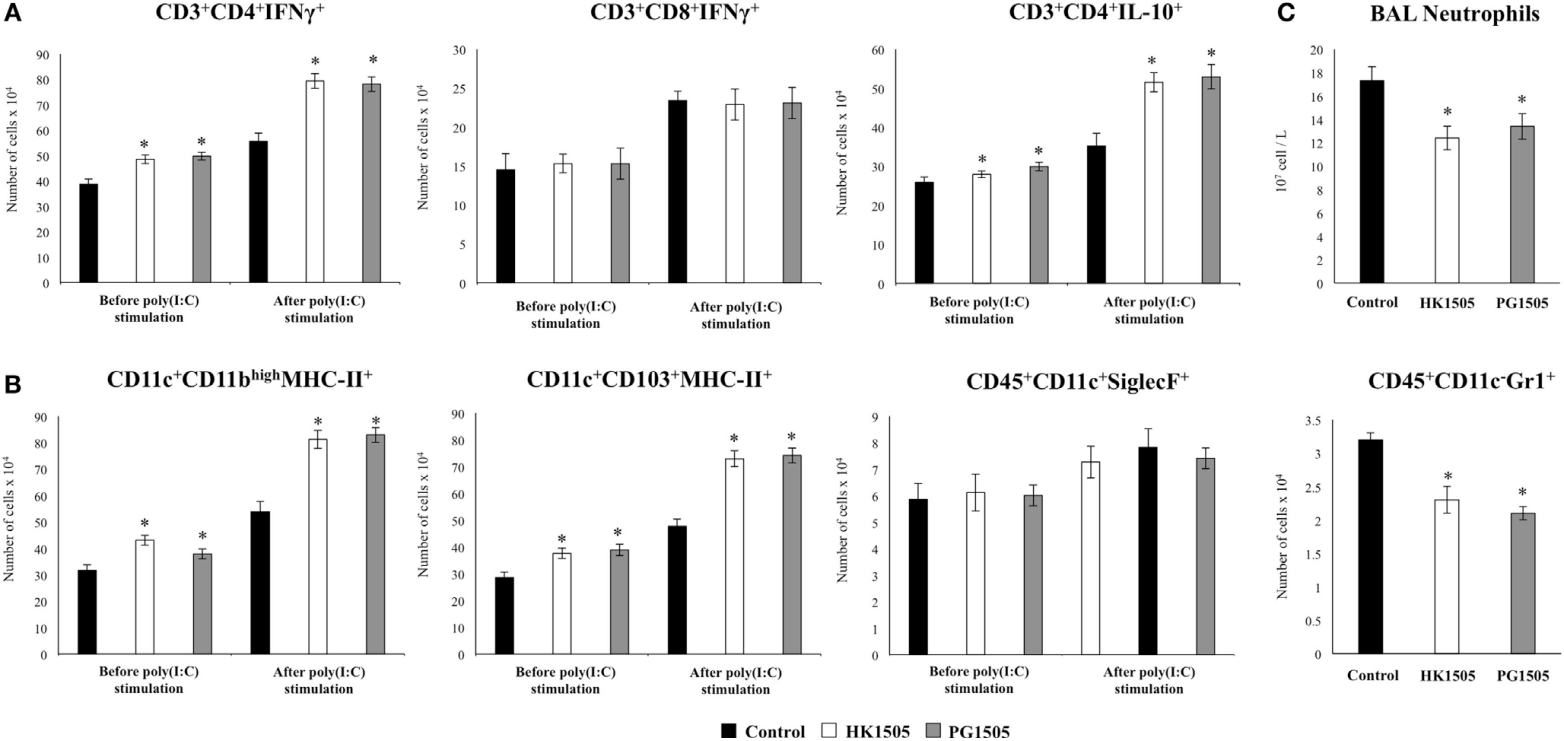
Effect of non-viable *Lactobacillus rhamnosus* CRL1505 (HK1505) and its peptidoglycan (PG1505) on respiratory immune cell populations before and after the nasal administration of the viral pathogen-associated molecular pattern poly(I:C). Infant mice were nasally primed with HK1505 or PG1505 during two consecutive days and then challenged with three once-daily doses of poly(I:C). Non-treated infant mice challenged with poly(I:C) were used as controls. Before and 2 days after poly(I:C) administration, the numbers of lung T cells **(A)** including CD3^+^CD4^+^IFN-γ^+^, CD3^+^CD4^+^IL-10^+^, and CD3^+^CD8^+^IFN-γ^+^ T lymphocytes, as well as antigen presenting cells **(B)** including MHC-II^+^CD11c^+^CD11b^low^CD103^+^ and MHC-II^+^CD11c^+^CD11b^high^CD103^−^ dendritic cells, and CD45^+^CD11c^+^SiglecF^+^ alveolar macrophages were determined by flow cytometry. In addition, bronchoalveolar lavages (BAL) neutrophils and lung CD45^+^CD11c^−^Gr1^+^ cells **(C)** were determined. The results represent data from three independent experiments. Significant differences between treated and control groups, *P < 0.05.

In order to evaluate the effect of HK1505 and PG1505 in the respiratory immune system of infant mice, we first determined the levels of different cytokines in BAL before poly(I:C) administration (Figure 2). Both, HL1505 and PG1505 treatments significantly increased the levels of BAL IFN-α, IFN-β, IFN-γ, IL-10, and IL-6. BAL concentrations of IFN-α in HK1505 mice and IL-6 in PG1505 mice were higher than the other experimental groups. No significant differences were found when the levels of TNF-α, MCP-1, and IL-8 of control mice were compared with mice receiving HK1505 or PG1505 (Figure 2). The treatments were also able to increase serum IFN-α, IFN-β, IFN-γ, IL-10, and IL-6, being both HK1505 and PG1505 equally effective to achieve this effect (Figure S2 in Supplementary Material). No differences were found in the levels of serum TNF-α, MCP-1, or IL-8 when comparing HK1505 and PG1505 with controls. We also evaluated the changes induced by nasally administered HK1505 and PG1505 in lung immune cells using flow cytometry. Nasal priming with HK1505 and PG1505 enhanced the number of CD3^+^CD4^+^IFN-γ^+^ and CD3^+^CD4^+^IL-10^+^ T cells in lungs while no effect was observed for CD3^+^CD8^+^IFN-γ^+^ T cells when compared to controls (Figure 3). In addition, three population of antigen presenting cells were studied in lungs: myeloid DCs (MHC-II^+^CD11c^+^CD11b^low^CD103^+^ and MHC-II^+^CD11c^+^CD11b^high^CD103^−^ cells) and alveolar macrophages (CD45^+^MHC-II^−^CD11c^+^SiglecF^+^). HK1505 and PG1505 significantly increased the number of both lung CD11c^+^CD11b^low^CD103^+^ and CD11c^+^CD11b^high^CD103^−^ DCs, while no quantitative changes were detected in CD45^+^CD11c^−^SiglecF^+^ macrophages (Figure 3) or CD45^+^Gr1^+^neutrophils (data not shown).

The respiratory immune response triggered by poly(I:C) in infant mice and the effect of the nasal priming with HK1505 and PG1505 in that response were next studied. As we described previously (13, 17), the nasal administration of poly(I:C) significantly increased respiratory levels of type I IFNs (IFN-α, IFN-β), IFN-γ, and pro-inflammatory cytokines and chemokines (TNF-α, IL-6, MCP-1, IL-8) in BAL of infant mice (Figure 2). Both HK1505 and PG1505 treatments significantly increased the levels of BAL IFN-α, IFN-β, IFN-γ, and TNF-α while they diminished the concentration of IL-6 and MCP-1 (Figure 2). In addition, IL-8 was not modified in HK1505 or PG1505 groups when compared to control mice (Figure 2). Poly(I:C) stimulation also induced an increase in the respiratory levels of IL-10 that were significantly higher in HK1505- or PG1505-treated mice when compared to controls (Figure 2). Lung immune cells populations were also evaluated in poly(I:C)-challenged mice (Figure 3). Poly(I:C) administration increased the numbers of CD3^+^CD4^+^IFN-γ^+^, CD3^+^CD4^+^IL-10^+^, CD3^+^CD8^+^IFN-γ^+^ T cells, and myeloid DCs (MHC-II^+^CD11c^+^CD11b^low^CD103^+^ and MHC-II^+^CD11c^+^CD11b^high^CD103^−^ cells) in lungs as we described previously (13, 17). In addition, we also observed an increase in alveolar macrophages (CD45^+^CD11c^+^SiglecF^+^) and neutrophils (CD45^+^Gr1^+^) after the challenge with poly(I:C) when compared to basal levels (Figure 3). Nasal priming with HK1505 or PG1505 increased the numbers of lung CD3^+^CD4^+^IFN-γ^+^, and CD3^+^CD4^+^IL-10^+^ T cells as well as MHC-II^+^CD11c^+^CD103^+^ and MHC-II^+^CD11c^+^CD11b^high^ DCs (Figure 3). No significant modification of the numbers of lung CD3^+^CD8^+^IFN-γ^+^ T cells or CD45^+^CD11c^+^SiglecF^+^ macrophages (Figure 3) were observed after HK1505 or PG1505 treatments when compared to controls. Significantly reduced numbers of neutrophils in BAL and lung were found in HK1505- or PG1505-treated mice when compared to controls (Figure 3).

### Nasally Administered Peptidoglycan from *L. rhamnosus* CRL1505 Improves Resistance against RSV Infection

**Figure 4:**
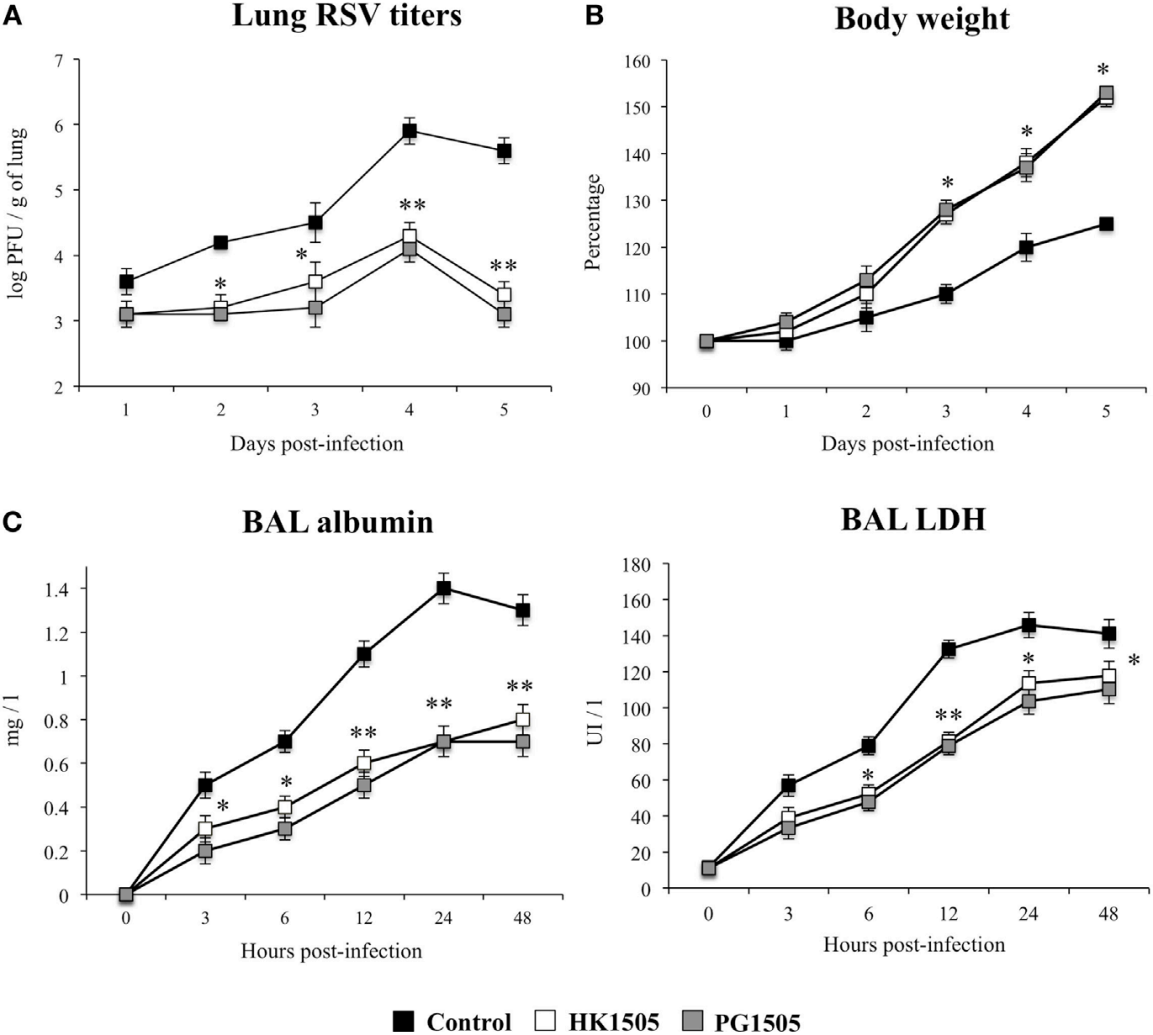
Effect of non-viable *Lactobacillus rhamnosus* CRL1505 (HK1505) and its peptidoglycan (PG1505) on the resistance to primary Respiratory Syncytial Virus (RSV) infection. Infant mice were nasally primed with HK1505 or PG1505 during two consecutive days and then challenged with RSV. Non-treated infant mice challenged with the viral pathogen were used as controls. Lung RSV titers **(A)**, changes in body weight **(B)**, and lactate dehydrogenase (LDH) activity and albumin concentrations in bronchoalveolar lavages (BAL) **(C)** were evaluated on different time points after the viral challenge. The results represent data from three independent experiments. Significant differences between treated and control groups *P < 0.05, **P < 0.01.

We next evaluated whether the changes induced by PG1505 in the respiratory immune system affected the outcome of RSV infection in infant mice as described previously for HK1505 (13). Therefore, infant mice were treated with HK1505 or PG1505 by the nasal route and then challenged with 10^6^ PFU of RSV (13, 16). Evaluation of viral loads of infected mice showed that RSV was present in lungs of all the experimental groups during the 5 days studied (Figure 4A). However, significantly lower viral titers were found in mice treated with HK1505 or PG1505 when compared to controls, being both treatments equally effective to reduce RSV replication in the respiratory tract. Moreover, HK1505 and PG1505 significantly improved the body weight gain during RSV infection when compared to controls (Figure 4B). The markers of lung tissue damage in RSV-infected mice showed that the viral infection induced a significant cellular damage and alveolar–capillary barrier alterations (Figure 4C). Both, BAL LDH and albumin concentrations were significantly lower in infant mice previously treated with HK1505 or PG1505 than in RSV-challenged control mice (Figure 4C).

### Nasally Administered Non-Viable *L. rhamnosus* CRL1505 and Its Peptidoglycan Improve Resistance to Secondary Pneumococcal Pneumonia after Poly(I:C) Treatment

As mentioned before, respiratory viral infections increase the susceptibly of secondary bacterial pneumonia in infants. Taking into consideration the beneficial effects of nasal HK1505 (13) or PG1505 on the respiratory antiviral innate immune response, we next addressed whether these treatments where able to increase the resistance of infant mice to secondary pneumococcal pneumonia. For this purpose, we first performed comparative studies of secondary pneumococcal infection in Swiss albino and BALB/c mice that are naturally susceptible and resistant to pneumococci, respectively. We found that BALB/c mice, which are highly susceptible to poly(I:C) and RSV, were a better animal model for studying poly(I:C)-pneumococcal or RSV-pneumococcal infection than Swiss albino mice (data not shown). In line with our findings, some works have established that BALB/c mice are suitable for studying poly(I:C)-induced respiratory damage and RSV-bacterial infections. Stark et al. (19) demonstrated that exposure of BALB/c mice to RSV significantly decreased *S. pneumoniae, Staphylococcus aureus*, or *Pseudomonas aeruginosa* clearance. In addition, the work showed that the effect of RSV infection on bacterial clearance was dependent on the mouse genetic background by performing experiments with C57BL/6J and FVBN/J mice, which are relatively resistant and susceptible to RSV infection respectively. C57BL/6J mice showed a modest change in pneumococcal clearance following RSV challenge, whereas FVBN/J mice showed a decrease in pneumococcal clearance following RSV.

**Figure 5:**
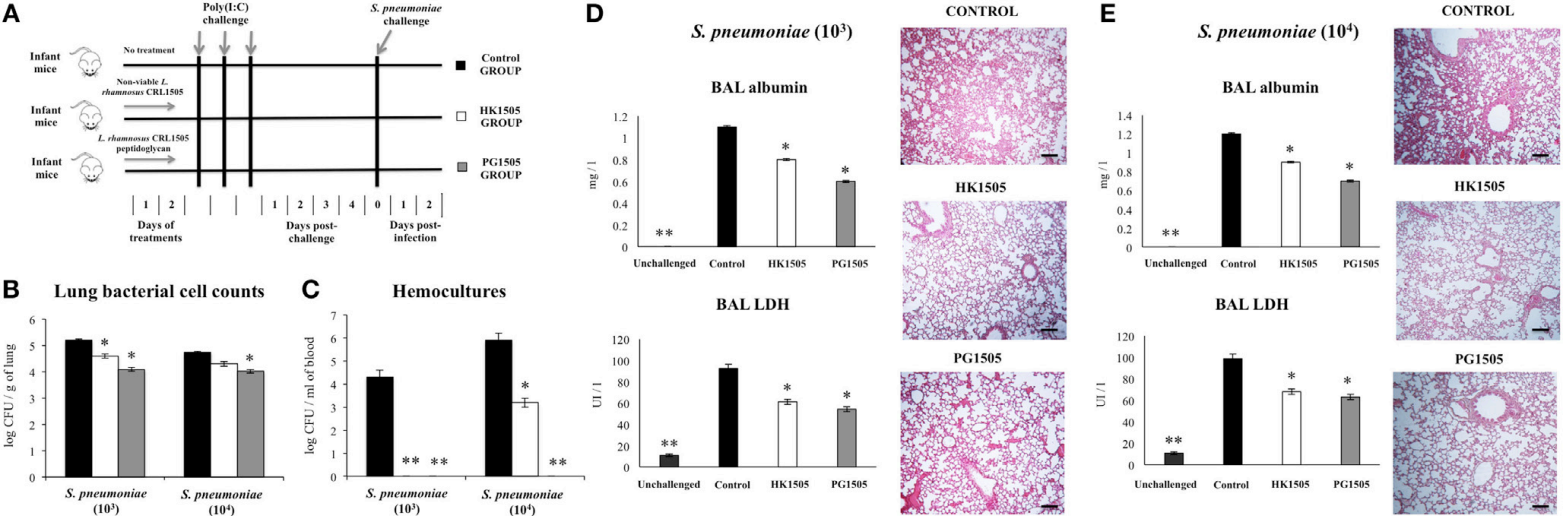
Effect of non-viable *Lactobacillus rhamnosus* CRL1505 (HK1505) and its peptidoglycan (PG1505) on the resistance to secondary pneumococcal pneumonia after the nasal administration of the viral pathogen-associated molecular pattern poly(I:C). Infant mice were nasally primed with HK1505 or PG1505 during two consecutive days, challenged with three once-daily doses of poly(I:C) and, infected with two different doses of *Streptococcus pneumoniae* 5 days after the last poly(I:C) administration **(A)**. Non-treated infant mice stimulated with poly(I:C) and challenged with *S. pneumoniae* were used as controls. Lung bacterial cells counts **(B)**, hemocultures **(C)**, lactate dehydrogenase (LDH) activity and albumin concentrations in bronchoalveolar lavages (BAL) **(D,E)**, and lung histopathological examination **(D,E)** were determined on day 2 post-pneumococcal challenge. Scale bar = 100 μm. The results represent data from three independent experiments. Significant differences between treated and control groups, *P < 0.05, **P < 0.01.

As shown in the experimental protocol illustrated in Figure 5A, mice were nasally primed with HK1505 or PG1505, stimulated with poly(I:C), and 5 days after the last poly(I:C) administration challenged with the respiratory bacterial pathogen *S. pneumoniae*. Pneumococcal colonization and bacteremia as well as lung tissue damage were evaluated on day 2 post-pneumococcal challenge. Two doses (10^3^ and 10^4^ cells) of *S. pneumoniae* were evaluated. Pneumococci were detected in lungs (Figure 5B) and blood (Figure 5C) of control infant mice for the two doses of the respiratory pathogen. HK1505 significantly reduced lung bacterial cell counts and the dissemination of *S. pneumoniae* into the blood in infant mice infected with 10^3^ pneumococcal cells while no significant differences in these parameters were found in animals infected with 10^4^ pneumococcal cells (Figure 5). Interestingly, PG1505 significantly reduced lung bacterial cell counts and the dissemination of the respiratory pathogen into the blood in infant mice infected with both 10^3^ and 10^4^ pneumococcal cells (Figures 5B,C). Evaluation of lung tissue injury showed that secondary pneumococcal pneumonia induced a significant cellular damage and alveolar–capillary barrier alterations as demonstrated by the significant higher levels of BAL LDH and albumin when compared to basal levels (Figures 5D,E). Lung histological examination revealed inflammatory cell recruitment around alveoli and blood vessels, focal hemorrhage, and a significant reduction of gas exchange spaces (Figures 5D,E). Biochemical markers and histology also showed that lung tissue injuries were comparable in mice infected with both 10^3^ and 10^4^ pneumococcal cells. BAL LDH and albumin concentrations were significantly lower in infant mice treated with HK1505 or PG1505 when compared to controls for both 10^3^ and 10^4^ pneumococcal cells (Figures 5D,E). Moreover, lung histology of HK1505- or PG1505-treated mice showed a significant reduction in the alterations of gas exchange spaces, hemorrhage, and inflammatory cells infiltration.

### Nasally Administered Non-Viable *L. rhamnosus* CRL1505 and Its Peptidoglycan Differentially Modulate the Immune Response Triggered by Secondary Pneumococcal Pneumonia

**Figure 6:**
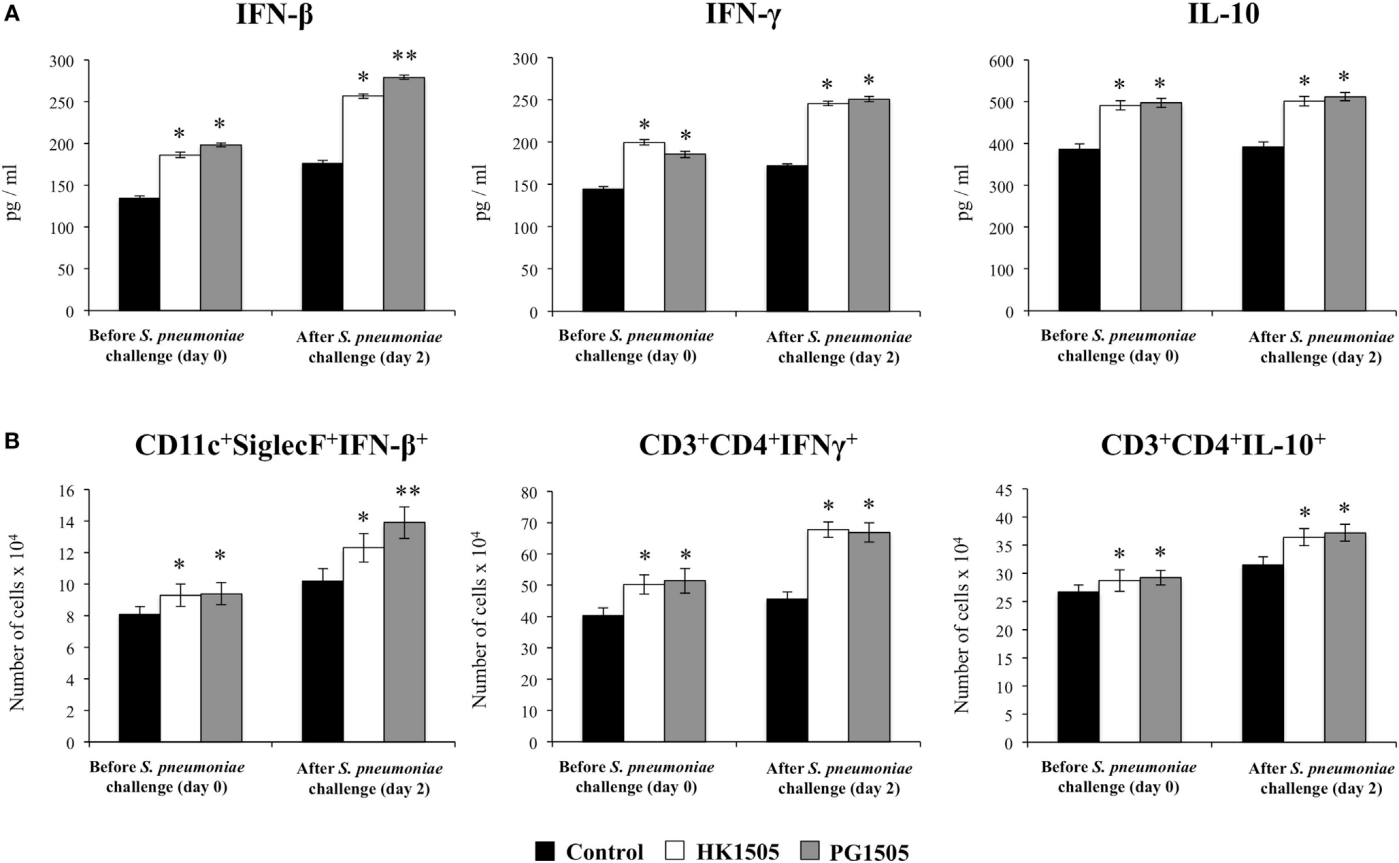
Effect of non-viable *Lactobacillus rhamnosus* CRL1505 (HK1505) and its peptidoglycan (PG1505) on the respiratory immune response to secondary pneumococcal pneumonia after the nasal administration of the viral pathogen-associated molecular pattern poly(I:C). Infant mice were nasally primed with HK1505 or PG1505 during two consecutive days, challenged with three once-daily doses of poly(I:C) and, infected with two different doses of *Streptococcus pneumoniae* 5 days after the last poly(I:C) administration. Non-treated infant mice stimulated with poly(I:C) and challenged with *S. pneumoniae* were used as controls. The levels of interferon (IFN)-β, IFN-γ, and interleukin (IL)-10 in bronchoalveolar lavages **(A)**, as well as the numbers of lung CD3^+^CD4^+^IFN-γ^+^, and CD3^+^CD4^+^IL-10^+^ T cells and CD45^+^CD11c^+^SiglecF^+^ alveolar macrophages **(B)** were determined before (day 0) and after (day 2) pneumococcal challenge. The results represent data from three independent experiments. Significant differences between treated and control groups, *P < 0.05, **P < 0.01.

Our previous results indicate that IFN-β, IFN-γ, and IL-10 are involved in the immunoregulatory effects of nasally administered immunobiotics (12). Moreover, as we described in Figure 2, the levels of these three cytokines were significantly increased in the respiratory tract of HK1505- or PG1505-treated mice. Therefore, we aimed to investigate the levels of IFN-β, IFN-γ, and IL-10 before (day 0) and after (day 2) the infection with *S. pneumoniae* as indicated in the experimental protocol of Figure 5A. The three cytokines were increased in HK1505 and PG1505 groups indicating that their elevated levels persisted up to 5 days of the last poly(I:C) administration (Figure 6A). Pneumococcal challenge further increase BAL IFN-β, IFN-γ, and IL-10 that were higher in HK1505- or PG1505-treated mice when compared to controls (Figure 6A). We also investigated the potential source of these cytokines within the immune cell populations of lungs. As we described previously, we found that IFN-γ and IL-10 were mainly produced by CD4^+^ T cells. In this work, we also demonstrated that IFN-β was mainly produced by alveolar macrophages and the CD45^−^ population of lung (probably respiratory epithelial cells) (data not shown). Similar to the results of cytokines’ levels, we found that lung CD3^+^CD4^+^IFN-γ^+^, and CD3^+^CD4^+^IL-10^+^ T cells as well as CD11c^+^SiglecF^+^IFN-β^+^ macrophages were significantly increased before and after the infection with *S. pneumoniae* in HK1505- or PG1505-treated mice when compared to control animals (Figure 6B). Of interest, both CD11c^+^SiglecF^+^IFN-β^+^ macrophages and BAL IFN-β levels were higher in the PG1505 group than in HK1505 mice (Figure 6).

**Figure 7:**
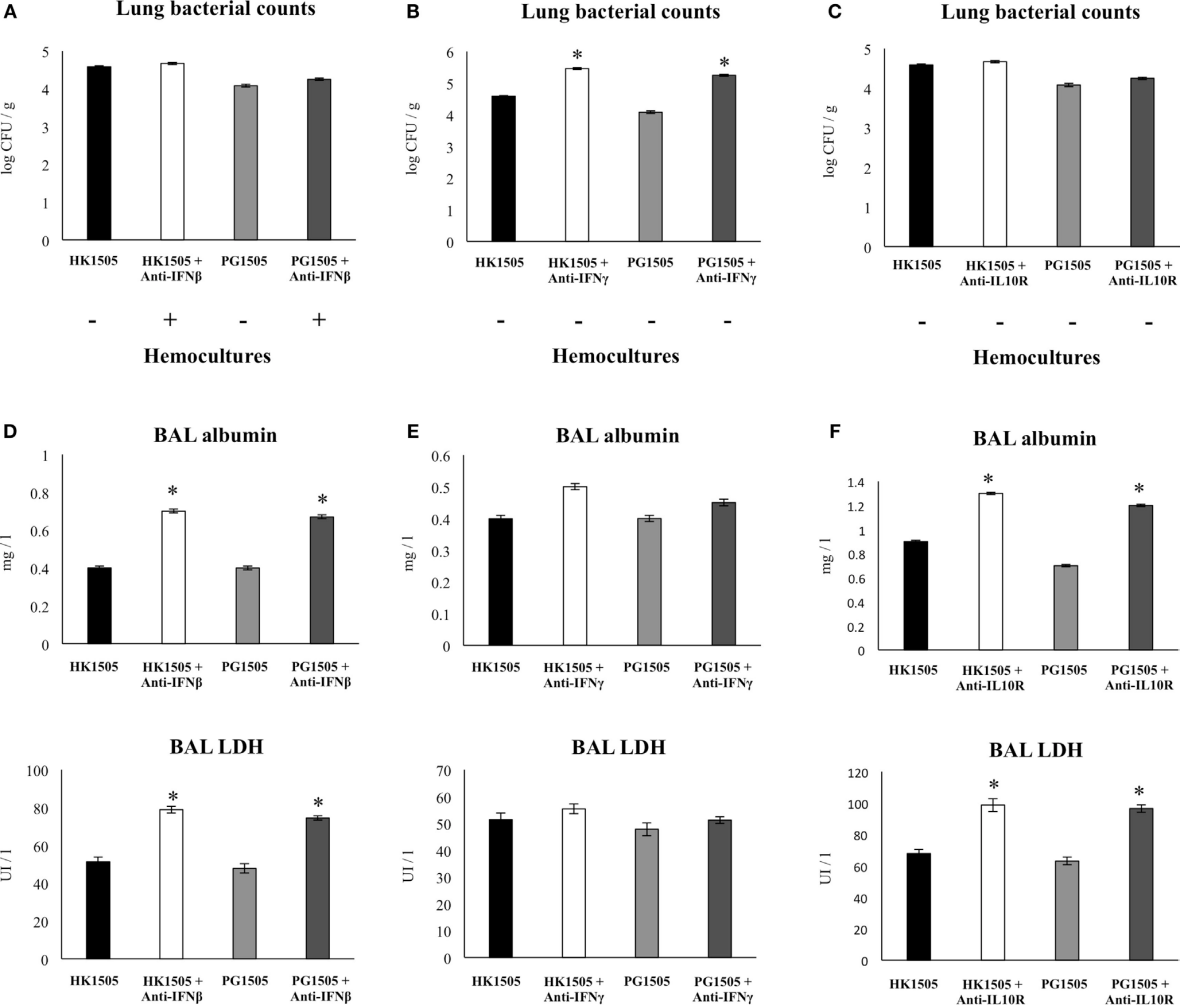
Role of interferon (IFN)-β, IFN-γ, and interleukin (IL)-10 in the immunomodulatory effect of non-viable *Lactobacillus rhamnosus* CRL1505 (HK1505) and its peptidoglycan (PG1505) on the resistance to secondary pneumococcal pneumonia after the nasal administration of the viral pathogen-associated molecular pattern poly(I:C). Infant mice were nasally primed with HK1505 or PG1505 during two consecutive days, and then challenged with three once-daily doses of poly(I:C). On days 2 and 4 after poly(I:C) challenge, mice were nasally treated anti-IFN-β, anti-IFN-γ, or anti-IL-10 receptor (IL-10R) blocking antibodies. Control HK1505 and PG1505 received isotype control antibodies. Twelve hours later mice were challenged with *Streptococcus pneumoniae*. Lung bacterial cells counts and hemocultures **(A–C)**, as well as lactate dehydrogenase (LDH) activity and albumin concentrations in bronchoalveolar lavages (BAL) **(D–F)** were determined on day 2 post-pneumococcal challenge. The results represent data from three independent experiments. Significant differences between treated and control groups, *P < 0.05.

In order to further evaluate the role of IFN-β, IFN-γ, and IL-10 in the immunoregulatory effect of HK1505 and PG1505 during secondary pneumococcal infection we used blocking anti-IFN-β, anti-IFN-γ, and anti-IL-10R antibodies as described in the experimental protocol of Figure S2 in Supplementary Material. Treatment of mice with anti-IFN-β significantly abolished the capacity of HK1505 and PG1505 to avoid pneumococcal dissemination into the blood although it had no effect on lung bacterial cell counts (Figure 7A). In addition, the ability of HK1505 and PG1505 to protect against pulmonary damage was lost with anti-IFN-β antibodies administration (Figure 7D). Treatment with anti-IFN-γ significantly abolished the capacity of HK1505 and PG1505 to reduce lung bacterial cell counts and had no effect on bacteremia (Figure 7B) or in the protection against lung tissue damage (Figure 7E). On the other hand, administration of anti-IL-10R to infant mice significantly abolished the capacity of HK1505 and PG1505 to reduce lung tissue injuries (Figure 7F), while they did not affect lung pneumococcal colonization or dissemination into the blood (Figure 7C).

### Nasally Administered Non-Viable *L. rhamnosus* CRL1505 and Its Peptidoglycan Improve Resistance to Secondary Pneumococcal Pneumonia after RSV Infection

**Figure 8:**
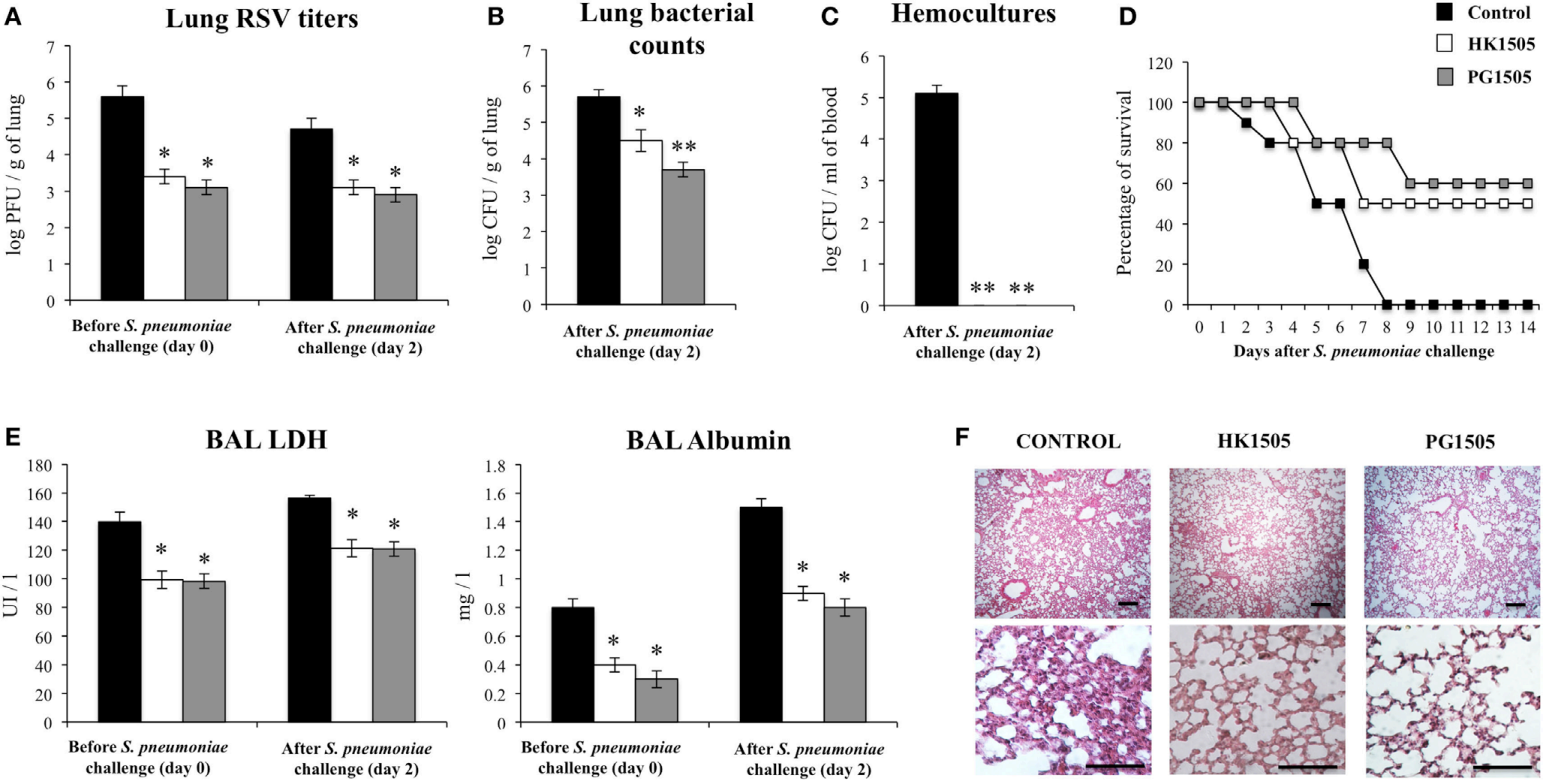
Effect of non-viable *Lactobacillus rhamnosus* CRL1505 (HK1505) and its peptidoglycan (PG1505) on the resistance to secondary pneumococcal pneumonia after the primary infection with Respiratory Syncytial Virus (RSV). Infant mice were nasally primed with HK1505 or PG1505 during two consecutive days, challenged with RSV and, infected with *Streptococcus pneumoniae* 5 days after the viral infection. Non-treated infant mice infected with RSV and challenged with *S. pneumoniae* were used as controls. Lung RSV titers **(A)**, lung bacterial cells counts **(B)**, hemocultures **(C)**, survival **(D)**, lactate dehydrogenase (LDH) activity, and albumin concentrations in bronchoalveolar lavages (BAL) **(E)**, and lung histopathological examination **(F)** were determined on day 2 post-pneumococcal challenge. Scale bar = 100 μm. The results represent data from three independent experiments. Significant differences between treated and control groups, **P < 0.05, **P < 0.01.

Finally, we aimed to evaluate whether HK1505 or PG1505 treatments were able to protect against secondary pneumococcal pneumonia after the infection of infant mice with RSV. For this purpose, mice were nasally primed with HK1505 or PG1505, infected with RSV, and 5 days after the infection they were challenged with 10^3^ cells of *S. pneumoniae*. Similar to the experiments performed with poly(I:C), pneumococcal colonization and bacteremia were evaluated on day 2 post-pneumococcal challenge. In addition, RSV titers as well as lung tissue damage were studied before (day 0) and after (day 2) the infection with *S. pneumoniae* (Figure 8). RSV was detected in the lungs of infected infant mice before and after pneumococcal infection (Figure 8A). In addition, pneumococci were detected in lungs (Figure 8B) and blood (Figure 8C) of control infant mice. Both, HK1505 and PG1506 significantly reduced RSV titers as well as lung bacterial cell counts and the dissemination of *S. pneumoniae* into the blood (Figures 8A–C), which was in line with the improved survival of HK1505- and PG1506-treated mice when compared to controls (Figure 8D). Of interest, PG1505 was more effective than HK1505 to diminish lung bacterial cell counts. When lung injury was studied, it was observed that the secondary pneumococcal pneumonia induced a significant increase of the BAL biochemical parameters that evaluate cellular damage and alveolar–capillary barrier alterations (Figure 8E). In addition, histological examination of lungs showed a significant reduction of gas exchange spaces, inflammatory cell recruitment, and focal hemorrhage (Figure 8F). HK1505 and PG1505 significantly reduced pulmonary damage as demonstrated by the diminished BAL LDH and albumin concentrations and the lower histological alterations when compared to controls (Figures 8E,F).

## Discussion

It was established that the mortality associated to respiratory viral infections is not due to the viral infection alone but instead, secondary bacterial pneumonia complicates many severe cases in infected hosts (1–3). Therefore, it is crucial to understand how respiratory viral infections alter the host’s respiratory microenvironment and the local innate immunity to benefit the establishment of secondary bacterial infections, in order develop preventive or therapeutic strategies aimed to protect against mortality. Considering the elevated incidence of viral infections and the high frequency of associated secondary bacterial infections that contribute to aggravate the health status, several approaches are been investigated for preventing or treating respiratory superinfections, including antibiotics and immunomodulatory drugs (20). To our knowledge, no study has evaluated the potential protective ability of immunobiotics or their immunomodulatory cellular fractions in the context of a secondary bacterial pneumonia. Therefore, we demonstrated here for the first time, that the nasal priming with peptidoglycan from the immunobiotic *L. rhamnosus* CRL1505 is able to improve the resistance of infant mice to primary RSV infection and secondary pneumococcal pneumonia.

The first conclusion that can be obtained from the data presented in this work is that peptidoglycan from immunobiotic *L. rhamnosus* CRL1505 preserves the immunomodulatory properties of the viable strain. We have demonstrated previously that the nasal priming with viable *L. rhamnosus* CRL1505 beneficially modulates the respiratory immune response triggered by TLR3 activation and increases the resistance to RSV infection (13). Similar to viable *L. rhamnosus* CRL1505, its purified peptidoglycan administered before the nasal challenge with three once-daily doses of poly(I:C) significantly increased the levels of BAL type I IFNs, especially IFN-β. Thus, PG1505 would enhance the expression of hundreds of IFNs-induced genes that counteract viral replication. This is in line with the improved resistance of PG1505-treated infant mice to the infectious challenge with RSV. Moreover, PG1505 differentially regulated the production of pro- and anti-inflammatory cytokines in response to TLR3 activation which are also important for the generation of an effective respiratory antiviral response. PG1505 improved the production of some cytokines (TNF-α, IL-10) while it reduced the levels of other pro-inflammatory factors (IL-6, MIP-1) indicating that the inflammatory response was differentially modulated. In fact, PG1505-treated mice showed an improved capacity to control RSV replication in the respiratory tract and a reduced inflammatory damage in the lung tissue. Resembling the effect of viable *L. rhamnosus* CRL1505 (13), PG1505 improved the numbers of CD3^+^CD4^+^IFN-γ^+^, MHC-II^+^CD11b^low^CD103^+^, and MHC-II^+^CD11b^high^CD103^−^ DCs as well as the respiratory levels of IFN-γ indicating the generation of a Th1 response, that is also involved in the immune protection against respiratory viral attack. These results indicate that peptidoglycan is a key bacterial component involved in the immunomodulatory and antiviral capacity of the immunobiotic strain *L. rhamnosus* CRL1505.

An important finding of this work is that both HK1505 and PG1505 were able to differentially modulate the response of infant mice to the secondary pneumococcal pneumonia produced after the challenge with poly(I:C) or RSV. Slight but still significant reduction of pneumococcal cell counts were found in the lungs of HK1505- or PG1505-treated mice when compared to controls. The effect of HK1505 and PG1505 in reducing lung pneumococcal cell counts was modest compared with our own previous studies. We had reported that the nasal administration of viable *Lactococcus lactis* NZ9000 to adult and infant mice reduced in more than 2 log *S. pneumoniae* cell counts in lungs (21). In addition, non-viable *L. casei* CRL431 (22), and non-viable *L. rhamnosus* CRL1505 or its peptidoglycan (15) allowed to immunocopromised malnourished mice to completely eliminate *S. pneumoniae* from lungs. Moreover, HK1505 and PG1505 evaluated in an infant mice model of primary pneumococcal infection also reduced in more than 2 log *S. pneumoniae* cell counts in lungs when compared to untreated controls (data not shown). Then, immunobiotics and their cellular fractions would be more efficient to improve resistance to primary than secondary pneumococcal pneumonia.

Despite the modest results obtained by measuring the burden of the pathogen in the respiratory tract, PG1505 treatment was able to significantly reduce lung tissue damage and bacterial dissemination into the blood stream. These findings are of importance because studies in clinical trials (5, 6) and animal models of RSV-*S. pneumoniae* superinfection (7, 8) showed that enhanced lung injuries and elevated levels of bacteremia are critical factors that determine the severity of infection and the rate of mortality. In fact, PG1505-treated infant mice showed a significant improvement of survival after superinfection with RSV and *S. pneumoniae*. As mentioned before, several modifications induced by respiratory viruses are involved in facilitating *S. pneumoniae* infection, including the destruction of the respiratory epithelium, ciliary dyskinesia, enhancement of adhesion factors, and alterations of the immune response (7–10). In relation to immunopathology, it was reported that sequential infection with RSV and *S. pneumoniae* induced a significantly greater inflammation with high levels of infiltrated neutrophils and elevated levels of pro-inflammatory factors in the lung compared with mice that were inoculated with each pathogen separately (19). It is possible to speculate that the protective effect of PG1505 would be exerted at different levels. (a) The improvement of antiviral response and the consequent reduction of RSV titers in the lung, would contribute to the reduction of respiratory epithelium damage. (b) A reduction *S. pneumoniae* adhesion by diminishing the expression of RSV G protein and adhesion molecules in respiratory epithelial cells. In relation to this point, although we have not evaluated the impact of PG1505 in the expression of adhesion molecules in the respiratory tract, our recent transcriptomic studies evaluating the effect of *L. rhamnosus* CRL1505 in poly(I:C) challenged intestinal epithelial cells showed that the immunobiotic strain is able to differentially modulate the expression of several adhesion molecules including selectins E and L as well as ICAM-1, and EPCAM (23). Therefore, evaluating the effect of PG1505 on the expression of relevant adhesion molecules in respiratory epithelial cells is an interesting topic for future investigations. (c) The differential modulation of the respiratory innate immune response that allow a reduction of RSV and *S. pneumoniae* replication with minimal inflammatory damage of lung tissue.

Our results suggest that pneumococcal growth in lungs, dissemination into blood, and inflammatory tissue damage are events which are not strictly coupled with each other. Moreover, they are regulated by different factors during the development of secondary pneumococcal pneumonia as became evident when experiment with blocking anti-IFN-β, anti-IFN-γ, and anti-IL-10R antibodies were performed. In line with our results, Damjanovic et al. (24) demonstrated in a mice model of IFV-*S. pneumoniae* superinfection that uncontrolled bacterial outgrowth and excessive inflammation are not strictly coupled events, and that both are contributors to deleterious lung immunopathology and death. In fact, the work reported that the use of a bacteriostatic antibiotic effectively improved clinical outcome by controlling pneumococcal replication but it failed to significantly diminish pulmonary immunopathology. On the other hand, the use of dexamethasone slightly reduced immunopathology while it had no impact on bacterial clearance. Interestingly, administration of dexamethasone in combination the bacteriostatic antibiotic significantly improved pulmonary immunopathology, bacterial elimination, and most importantly the survival of infected mice (24). All these results together suggest that effective intervention strategies for respiratory superinfections need to involve an efficient control of pathogens growth and aberrant host inflammatory responses.

We found that at least three cytokines are involved in the immunomodulatory protective effect of HK1505 and PG1505 in the respiratory superinfection: IL-10, IFN-β, and IFN-γ. Our results indicated that IL-10 was involved in the protection against inflammatory damage while IFN-γ participated in the reduction of pneumococcal growth in the lungs. This is in line with our previous findings demonstrating that the protection of infant mice against poly(I:C) or RSV challenge induced by viable *L. rhamnosus* CRL1505 was dependent of both IL-10 and IFN-γ (16). Moreover, as observed for viable *L. rhamnosus* CRL1505 (16), both HK1505 and PG1505 improved the numbers of CD3^+^CD4^+^IFN-γ^+^, and CD3^+^CD4^+^IL-10^+^ T cells in the lungs of infant mice. These immune cell populations remained significantly elevated when compared to controls after pneumococcal challenge, indicating their participation in the protection against secondary bacterial pneumonia.

In addition, we showed that IFN-β was involved in the protection against lung tissue injury as well in the control of pneumococcal dissemination into the blood. The lung epithelial–endothelial layer is an important barrier in pneumococcal pathogenesis, since its alteration results in serious complications such as bacteremia and meningitis that are associated with high mortality. Some works have demonstrated an important role of IFN-β in the control of pneumococcal infection and invasiveness. Studies in IFNAR1 or IFN-β knockout mice showed that the abolishment of the IFN-β-IFNAR1 pathway increased nasopharyngeal carriage and enhanced mortality upon pneumococcal infection (25, 26). It was also demonstrated by LeMessurier et al. (27) that IFN-β increase the expression of tight junction proteins and reduce PAF receptor expression, which correlates with diminished pneumococcal invasion and transmigration of pulmonary epithelial and endothelial cells. In addition, intranasal administration of IFN-β was found to protect mice against the development of pneumococcal systemic disease (27). The role of IFN-β in secondary pneumococcal pneumonia has been also studied. No significant differences in *S. pneumoniae* counts in the lungs of IFNAR1^−/−^ and IFNAR1^+/+^ mice were observed after pneumococcal challenge. However, pneumococci were observed earlier and at higher numbers in blood samples of IFNAR1^−/−^ mice compared to wild-type animals (27). We demonstrated here that the improvement of IFN-β by PG1505 treatment would be related to the enhancement of CD11c^+^SiglecF^+^IFN-β^+^ alveolar macrophages. This is in line with studies demonstrating that alveolar macrophages are the main producers of type I IFNs during pulmonary viral infections (28, 29). However, it should be noted that the primary type I IFN response is mediated not only by alveolar macrophages but also by epithelial cells of the respiratory tract (25, 27). Therefore, respiratory epithelial cells may represent promising cellular target for future mechanistic studies in order to explain the protective effect of PG1505.

Our results demonstrated that the modulation of the three cytokines (IFN-β, IFN-γ, and IL-10) are necessary to achieve protection against secondary pneumococcal pneumonia. Therefore, an appropriate balance between pro- and anti-inflammatory factors would be necessary in order to obtain full protection without affecting lung structure and function. In line with this statement, some works have demonstrated that the excessive production of one particular cytokine have negative consequences in the outcome of respiratory infections. It was showed that excessive production of IFN-β during viral infection induce impaired neutrophils responses due to inadequate production of neutrophil chemoattractants (30). Although neutrophils infiltration has been linked to lung tissue damage, the phagocytic and bactericidal activities of these cells are necessary for the initial control of pneumococci. In addition, it was observed that neutralization of IFN-γ in IFV-infected mice considerably diminished bacterial susceptibility and improved survival after secondary pneumococcal pneumonia (31). This effect was explained by the inhibition of bacterial phagocytosis by the excessive production of IFN-γ. Data obtained from mice infected with IFV and *S. pneumoniae* demonstrated that the exaggerated production of IL-10 was associated with increased pneumococcal colonization and enhanced mortality (32). The work showed that the treatment of mice with blocking anti-IL-10 antibodies before secondary pneumococcal infection resulted in reduced bacterial cell counts in lungs and prolonged survival (32). Moreover, it can be anticipated that other cytokines are involved in susceptibility to secondary bacterial respiratory infections. In this regard, recent findings of Chen et al. (33) demonstrated that the anti-inflammatory cytokine IL-35 contributes to the decreased resistance to secondary pneumococcal pneumonia. The work reported that IFV infection induced a high expression of IL-35 in the respiratory tract and that secondary pneumococcal infection leaded to a synergistic production of this cytokine. Lung enhancement of IL-35 inhibited the early immune response against *S. pneumoniae*.

In summary, we demonstrated that the nasal priming with peptidoglycan from *L. rhamnosus* CRL1505 differentially modulates the respiratory innate antiviral immune response triggered by TLR3 activation in infant mice, improving the resistance to primary RSV infection, and secondary pneumococcal pneumonia. In association with the protection against RSV-pneumococcal superinfection, we found that peptidoglycan from *L. rhamnosus* CRL1505 significantly improved lung CD3^+^CD4^+^IFN-γ^+^, and CD3^+^CD4^+^IL-10^+^ T cells as well as CD11c^+^SiglecF^+^IFN-β^+^ alveolar macrophages with the consequent increases of IFN-γ, IL-10, and IFN-β in the respiratory tract. Our results also demonstrated that the increase of the three cytokines is necessary to reduce the severity of the respiratory superinfection since each of them are involved in different aspect of the secondary pneumococcal pneumonia that have to be controlled in order to reduce the severity of the infectious disease: lung pneumococcal colonization, bacteremia, and inflammatory-mediated lung tissue injury. The findings of this work will lead us in new directions to explore the molecular mechanisms *via* which peptidoglycan from *L. rhamnosus* CRL1505 interacts with immune and non-immune cells of the respiratory tract and to investigate whether its immunomodulatory properties are unique or common to peptidoglycans of other immunobiotic strains with antiviral capabilities.

## Ethics Statement

This study was carried out in strict accordance with the recommendations in the Guide for the Care and Use of Laboratory Animals of the Guidelines for Animal Experimentation of CERELA and all efforts were made to minimize suffering.

## Author Contributions

SA, HK, and JV designed the study and wrote the manuscript. PC, PK, HZ, AT, AK, and SS did the laboratory work. PC, PK, SS, and JV performed statistical analysis. SS, SA, HK, and JV contributed to data analysis and interpretation. All authors read and approved the manuscript.

## Conflict of Interest Statement

The authors declare that the research was conducted in the absence of any commercial or financial relationships that could be construed as a potential conflict of interest.

## Supplementary Material

Figure S1 Role of interferon (IFN)-β, IFN-γ, and interleukin (IL)-10 in the immunomodulatory effect of non-viable *Lactobacillus rhamnosus* CRL1505 (HK1505) and its peptidoglycan (PG1505) on the resistance to secondary pneumococcal pneumonia after the nasal administration of the viral pathogen-associated molecular pattern poly(I:C). Infant mice were nasally primed with HK1505 or PG1505 during two consecutive days, and then challenged with three once-daily doses of poly(I:C). On days 2 and 4 after poly(I:C) challenge, mice were nasally treated anti-IFN-β, anti-IFN-γ, or anti-IL-10 receptor blocking antibodies. Control HK1505 and PG1505 received isotype control antibodies. Twelve hours later mice were challenged with *Streptococcus pneumoniae*.

Figure S2 Effect of non-viable *Lactobacillus rhamnosus* CRL1505 (HK1505) and its peptidoglycan (PG1505) on blood cytokines. HK1505 or PG1505 were nasally administered to infant mice during two consecutive days. Non-treated infant mice were used as controls. Levels of tumor necrosis factor (TNF)-α, interferon (IFN)-α, IFN-β, IFN-γ, interleukin (IL)-6, IL-8, IL-10, and monocyte chemoattractant protein-1 (MCP-1) were determined in bronchoalveolar lavages (BAL) **(A)** and serum **(B)**. The results represent data from three independent experiments. Significant differences between treated and control groups, *P < 0.05, ***P* < 0.01.
