# Supplementary figures and images for: Peptidoglycan from Immunobiotic Lactobacillus rhamnosus Improves Resistance of Infant Mice to Respiratory Syncytial Viral Infection and Secondary Pneumococcal Pneumonia
Source: Front Immunol. 2017 Aug 10;8:948. doi: 10.3389/fimmu.2017.00948 (PMC5554128; doi:10.3389/fimmu.2017.00948)

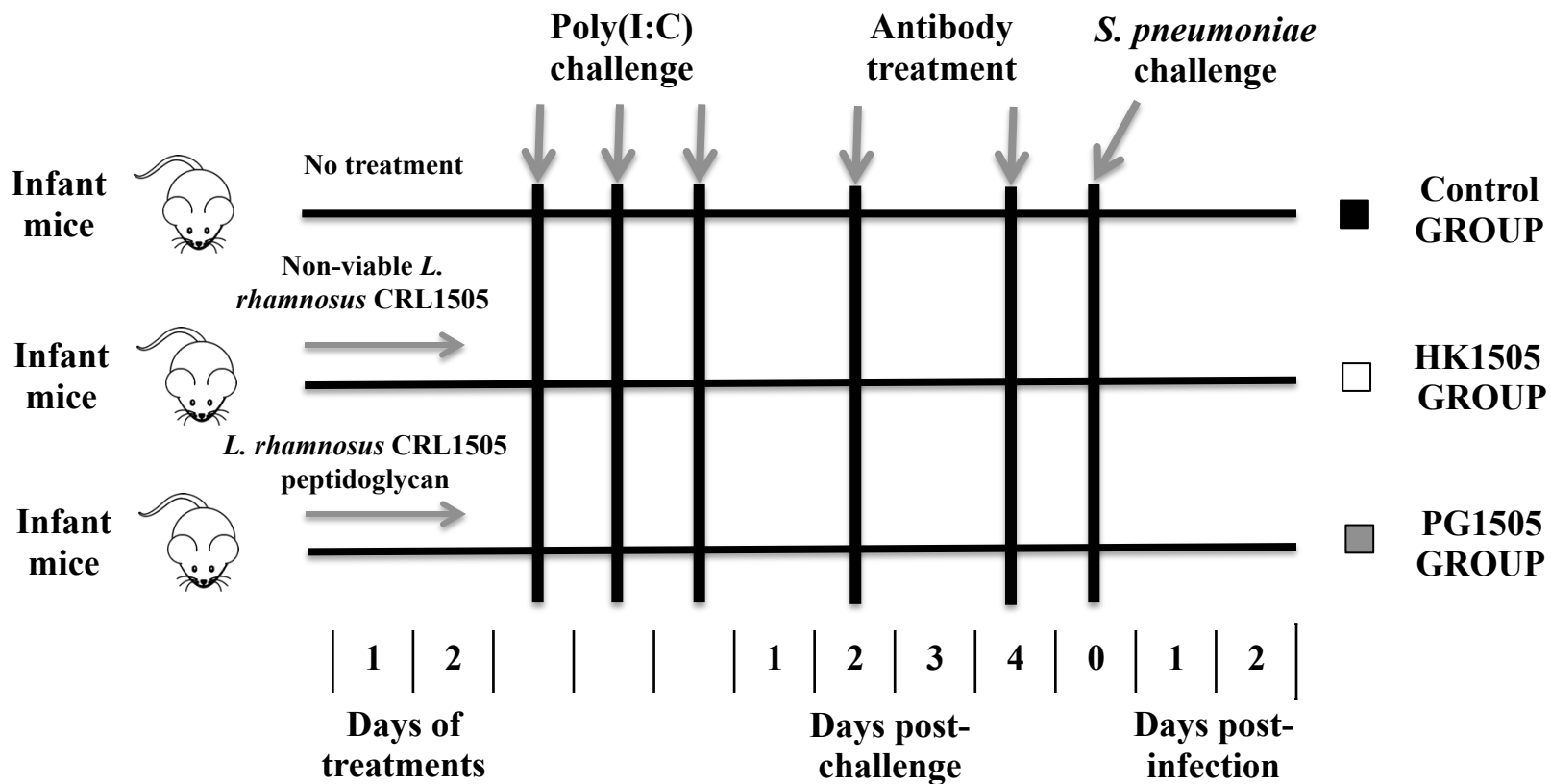

Supplement: Figure S1 — Role of interferon (IFN)-β, IFN-γ, and interleukin (IL)-10 in the immunomodulatory effect of non-viable Lactobacillus rhamnosus CRL1505 (HK1505) and its peptidoglycan (PG1505) on the resistance to secondary pneumococcal pneumonia after the nasal administration of the viral pathogen-associated molecular pattern poly(I:C). Infant mice were nasally primed with HK1505 or PG1505 during two consecutive days, and then challenged with three once-daily doses of poly(I:C). On days 2 and 4 after poly(I:C) challenge, mice were nasally treated anti-IFN-β, anti-IFN-γ, or anti-IL-10 receptor blocking antibodies. Control HK1505 and PG1505 received isotype control antibodies. Twelve hours later mice were challenged with Streptococcus pneumoniae. [file Image_1.PDF]

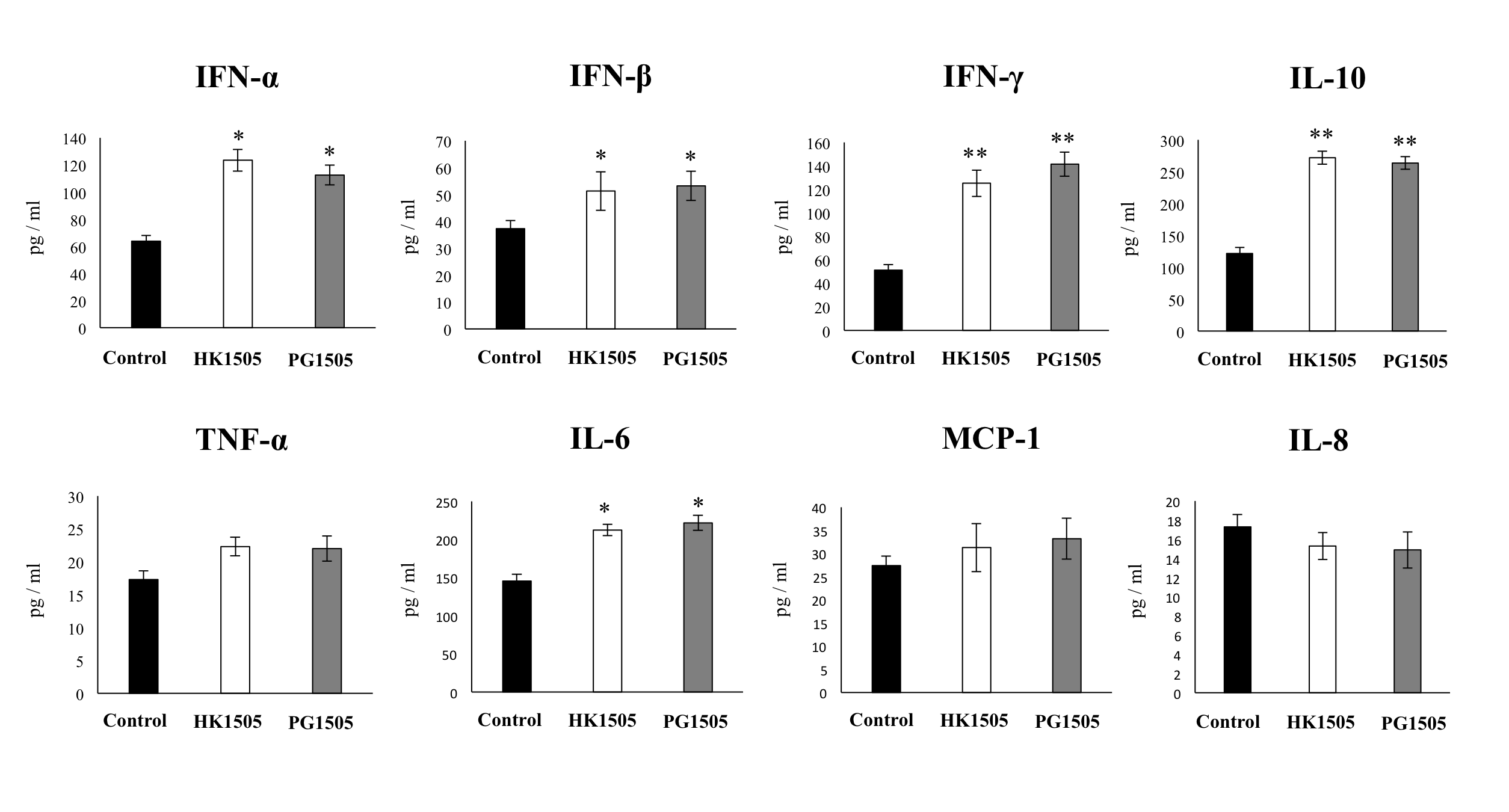

Supplement: Figure S2 — Effect of non-viable Lactobacillus rhamnosus CRL1505 (HK1505) and its peptidoglycan (PG1505) on blood cytokines. HK1505 or PG1505 were nasally administered to infant mice during two consecutive days. Non-treated infant mice were used as controls. Levels of tumor necrosis factor (TNF)-α, interferon (IFN)-α, IFN-β, IFN-γ, interleukin (IL)-6, IL-8, IL-10, and monocyte chemoattractant protein-1 (MCP-1) were determined in bronchoalveolar lavages (BAL) (A) and serum (B). The results represent data from three independent experiments. Significant differences between treated and control groups, *P < 0.05, **P < 0.01. [file Image_2.TIF]
